# Dosimetric analysis of ^123^I, ^125^I and ^131^I in thyroid follicle models

**DOI:** 10.1186/s13550-014-0023-9

**Published:** 2014-06-11

**Authors:** Anders Josefsson, Eva Forssell-Aronsson

**Affiliations:** 1 Department of Radiation Physics, Institute of Clinical Sciences, Sahlgrenska Cancer Centre, Sahlgrenska Academy at the University of Gothenburg, Gothenburg 413 45, Sweden

**Keywords:** Monte Carlo, MCNPX, S value, MIRD, Man, Rat, Mouse, Thyroid gland

## Abstract

**Background:**

Radioiodine is routinely used or proposed for diagnostic and therapeutic purposes: ^123^I, ^125^I and ^131^I for diagnostics and ^125^I and ^131^I for therapy. When radioiodine-labelled pharmaceuticals are administered to the body, radioiodide might be released into the circulation and taken up by the thyroid gland, which may then be an organ at risk. The aim of this study was to compare dosimetric properties for ^123^I, ^125^I and ^131^I in previously developed thyroid models for man, rat and mouse.

**Methods:**

Dosimetric calculations were performed using the Monte Carlo code MCNPX 2.6.0 and nuclear decay data from ICRP 107. Only the non-radiative transitions in the decays were considered. The *S* value was determined for the cell nuclei in species-specific thyroid follicle models for mouse, rat and man for different spatial distributions of radioiodine.

**Results:**

For the species-specific single follicle models with radioiodine homogeneously within the follicle lumen, the highest *S* value came from ^131^I, with the largest contribution from the β particles. When radioiodine was homogeneously distributed within the follicle cells or the follicle cell nucleus, the highest contribution originated from ^125^I, about two times higher than ^123^I, with the largest contribution from the Auger electrons. The mean absorbed dose calculated for our human thyroid multiple follicle model, assuming homogenous distribution of for ^123^I, ^125^I, or ^131^I within the follicle lumens and follicle cells, was 9%, 18% and 4% higher, respectively, compared with the mean absorbed dose according to Medical Internal Radiation Dose (MIRD) formalism and nuclear decay data. When radioiodine was homogeneously distributed in the follicle lumens, our calculations gave up to 90% lower mean absorbed dose for ^125^I compared to MIRD (20% lower for ^123^I, and 2% lower for ^131^I).

**Conclusions:**

This study clearly demonstrates the importance of using more detailed dosimetric methods and models than MIRD formalism for radioiodine, especially ^123^I and ^125^I, in the thyroid. For radioiodine homogeneously distributed in the follicle lumens our calculations for the human multiple follicle models gave up to 90% lower mean absorbed dose compared with MIRD formalism.

## Background

Radioiodine has been used clinically for about 70 years, both for diagnosis (^123^I, ^125^I and ^131^I) and therapy (^125^I and ^131^I) of various diseases. Radioiodine is to a high extent accumulated in the thyroid gland and metabolically built into the thyroid hormones, and the biokinetics of radioiodine is used for measurement of thyroid function [[1]]. ^131^I, as iodide, is therefore routinely used for diagnosis and treatment of various thyroid disorders such as thyrotoxicosis (hyperthyroidism) and thyroid cancer [[1]-[3]]. Lately, ^131^I has sometimes been replaced by ^123^I as iodide for diagnostic purposes to avoid the thyroid stunning phenomenon [[4],[5]]. Radioiodine bound to specific vector molecules is also widely used. For example, ^131^I- and ^123^I-MIBG are used for scintigraphy and ^131^I- and ^125^I-MIBG for treatment of various neuroendocrine tumours [[6],[7]], ^123^I-receptor ligands for brain scintigraphy [[8]], ^125^I methylene blue for sentinel node localization [[9]] and ^131^I-labelled monoclonal antibodies for treatment of lymphoma [[10]]. When, radioiodine labelled pharmaceuticals are administered to the body, radioiodide might be released into the circulation, e.g. by enzymatic reactions by dehalogenases in tissues, and taken up by the thyroid gland [[11]]. For radiopharmaceuticals or tracers labelled with radioiodine, the thyroid is thus an organ at risk.

Furthermore, ^131^I is a radionuclide of importance in nuclear accidents, where it is a rest product from the nuclear fission process in nuclear energy plants. After the Chernobyl accident in 1986, contamination with ^131^I (and other short-lived isotopes such as ^132^I and ^133^I) led to an increased incidence of differentiated thyroid cancers in children but not in adults, with a higher incidence with lower age [[12]–[15]].

There is thus a need for accurate dosimetric calculations of the absorbed dose for both patients examined or treated with radioiodine, personnel handling radioiodine and for personnel and the general population in case of accidental exposure to radioiodine.

Dosimetric estimations using the MIRD formalism is mostly utilised due to its simplicity, and the use of mean absorbed dose is of interest if the radionuclides and the energy deposited are homogeneously distributed within each organ/tissue. This assumption is adequate as long as the range of the emitted particles is long compared to the size of the cells. For radionuclides emitting particles with shorter range, e.g. Auger and internal conversion electrons, non-uniform distribution within an organ/tissue will give heterogeneous absorbed dose distribution, and more detailed dosimetric approaches are clearly needed.

The physical properties differ between these radioiodine isotopes. For ^131^I, emitting relatively high-energy β particles with a range up to 2 mm in tissue [[16]], the energy distribution will be relatively homogeneous within the thyroid, less dependent of the radionuclide distribution, while for ^125^I, emitting cascades of Auger electrons with ranges from a few nanometres up to around 23 μm in tissue, the energy deposition within the thyroid gland will be more heterogeneous, dependent of the distribution of the radionuclide, electrons emitted, half-life, and the amount of photons emitted [[17],[18]].

To be able to determine more detailed dosimetric parameters for the thyroid cells from heterogeneously distributed radioiodine isotopes in the thyroid tissue, thyroid tissue models are needed. We have recently published thyroid models for man, but also for mouse and rat, and performed microdosimetric studies of the α particle emitting radiohalogen ^211^At, demonstrating the importance of detailed dosimetry for the thyroid [[19]]. A few thyroid models for radioiodine dosimetry have been previously published for normal and thyrotoxic thyroid follicles [[20]-[24]]. To our knowledge, few dosimetric studies have been published demonstrating the dosimetric properties of these radioiodine nuclides in these models.

The aim of this study was to compare dosimetric calculations for ^123^I, ^125^I and ^131^I using the general purpose Monte Carlo radiation transport code MCNPX 2.6.0 [[25]] with nuclear decay data from ICRP 107 [[26]] and the recently developed thyroid models for man, rat and mouse [[19]].

## Methods

### The radioiodine isotopes ^123^I, ^125^I and ^131^I

Nuclear decay data from ICRP 107 for ^123^I, ^125^I and ^131^I were used in all the dosimetric calculations, if not stated otherwise [[26]]. Only the non-radiative (NR) transitions from Auger and Coster-Kronig electrons (denoted AE in the rest of the paper), internal conversion electrons (CE) and β particles were considered. The conventional AE spectrum was used, and AE with initial kinetic energies <1 keV were assumed to be fully absorbed within the source volume.

^123^I has a half-life of 13 h and decays by electron capture (EC) via ^123^Te (half-life 6.0 × 10^14^ years and yield 99.9996%) or ^123m^Te (half-life 119 days and yield 4.4 × 10^-5^%) to stable ^123^Sb. ^123^I emits CE with 246 possible initial kinetic energies and on average 14 AE per decay. The mean emitted energy per decay for AE with initial kinetic energies <1 keV is 1.25 keV [[26]].

^125^I has a half-life of 59 days and decays by EC to stable ^125^Te. ^125^I emits CE with six possible initial kinetic energies and on average 23 AE per decay. The mean emitted energy per decay for AE with initial kinetic energies <1 keV is 2.11 keV [[26]].

^131^I has a half-life of 8.0 days and decays by emission of β particles directly or via ^131m^Xe (half-life 11.8 days and yield 1.18%) to stable ^131^Xe. The full energy spectrum for the emission of β particles was considered, including all six independent transition spectra. ^131^I emits CE with 108 possible initial kinetic energies and on average 0.7 AE per decay. The mean emitted energy per decay for AE with initial kinetic energies <1 keV is 78.3 eV [[26]]. The contribution from ^131m^Xe was not considered in the dosimetric calculations.

### Monte Carlo calculations

Calculations were performed using the general purpose Monte Carlo radiation transport code MCNPX 2.6.0 [[25]] on a MacBook Pro with a 2.66 GHz Intel Core 2 Duo processor with Mac OS X version 10.6.8 (Apple Incorporated, Cupertino, CA, USA). The tally *F8 was used, which gives the energy imparted to the target volume. The default settings in MCNPX were used for the sampling frequency, and the cutoff energy was 1 keV. For each geometric setup, 1 to 5 × 10^7^ histories were simulated, depending on geometry, distribution and NR transition. The default particle transport physics were used in the electron transport calculations, which included multiple scattering, straggling for electron energy loss and generation of secondary electrons [[27]].

### Single thyroid follicle model

The single thyroid follicle model used has previously been described [[19]]. Briefly, the model consists of a single layer of thyroid follicular cells surrounding a spherical follicle lumen (diameter 10 to 500 μm). The follicle cells have a thickness of 6, 8 or 10 μm with a centrally located spherical nucleus with diameters of 4, 6 and 8 μm, respectively. For the species-specific models, the follicle lumen diameter, follicle cell thickness and nucleus diameter are: (1) 50, 6 and 4 μm for mouse, (2) 70, 8 and 6 μm for rat and (3) 150, 10 and 8 μm for man, respectively. All the follicle models were assumed to consist of liquid water with unit density (1.0 g/cm^3^). The radioiodine distributions investigated were (A) homogeneous distribution within the follicle lumen, (B) homogeneous distribution on concentric spherical surfaces in the follicle lumens, (C) homogeneous distribution within the follicle cells and (D) homogeneous distribution within the follicle cell nuclei. In all simulations, the targets were the six follicular cell nuclei symmetrically positioned on the Cartesian axes, and the result was the average value for these targets (*cf.* [[19]]).

### Multiple thyroid follicle models

A multiple thyroid follicle model was used to calculate the contribution from surrounding layers of follicles to the follicle cell nuclei in a centrally placed follicle, based on the previously published multiple follicle model [[19]]. Calculations were performed for the models of mouse, rat and man. The neighbouring follicles were modelled as one surrounding layer of follicle cells, one outer layer simulating the follicle lumens with the respective thickness: (1) 6 and 50 μm for the mouse, (2) 8 and 70 μm for the rat and (3) 10 and 150 μm for the human model and another surrounding layer of follicle cells. The number of surrounding follicle layers that contributed depended on the species and radioiodine isotopes and was 2, 1 and 8 for ^123^I, ^125^I and ^131^I, respectively, in the human model.

In this model, two radioiodine distributions were used: (E) homogeneous distribution within the surrounding follicle lumens and (F) homogeneous distribution within the surrounding follicle cells. The targets were the six follicle cell nuclei in the central follicle, similar to the single follicle model (*cf.* [[19]]).

### Dosimetric parameters

MIRD formalism was used to calculate the mean absorbed dose, *D*(*r*_*T*_)_,_ using the expression

(1) $$D\left(r_{T}\right)=\frac{\tilde{A}\left(r_{S},T_{D}\right)}{M\left(r_{T}\right)}\cdot \sum_{i}E_{i}\cdot Y_{i}\cdot \phi \left(r_{T}\leftarrow r_{S}\right)\;\left[\mathrm{Gy}\right]$$

where $\tilde{A}\left(r_{S},T_{D}\right)$ is the time-integrated activity (according to MIRD pamphlet no. 21, previously named cumulated activity in MIRD primer), in the source volume in units of Bq⋅s, *M*(*r*_*T*_) is the mass of the target volume in units of kilogrammes [[28],[29]]. *E*_*i*_ ⋅ *Y*_*i*_, is the mean electron energy per transformation (26.7, 16.5 and 191.2 keV for ^123^I, ^125^I and ^131^I, respectively according to web published MIRD decay data [[30]]), and *ϕ*(*r*_*T*_ ← *r*_*S*_) is the absorbed fraction in the target volume for the emitted electrons per nuclear transformation, and was assumed to be unity for ^123^I, ^125^I and ^131^I in the thyroid gland of man.

The cumulative specific activity, $\tilde{C}\left(r_{S},T_{D}\right)$_,_ is expressed as

(2) $$\tilde{C}\left(r_{S},T_{D}\right)=\frac{\tilde{A}\left(r_{S},T_{D}\right)}{M\left(r_{T}\right)}\;\left[\mathrm{Bq}\cdot s/\mathrm{kg}\right]$$

To achieve a calculated mean absorbed dose of 1 Gy to the thyroid gland according to MIRD formalism (Equation 1), a $\tilde{C}\left(r_{S},T_{D}\right)\;$ equal to 234, 378 and 32.6 TBq⋅s/kg was needed for ^123^I, ^125^I and ^131^I, respectively.

The mean absorbed dose can also be calculated using the expression

(3) $$D\left(r_{T}\right)=\tilde{A}\left(r_{S},T_{D}\right)\cdot S\left(r_{T}\leftarrow r_{S}\right)\;\left[\mathrm{Gy}\right]$$

where *S*(*r*_*T*_ ← *r*_*S*_) is the mean absorbed dose per unit cumulated activity in the source volume in units of Gy/Bq⋅s [[29]] and will be denoted as the S value in the continuation of the text.

## Results

### Single follicle model

#### Radioiodine in species-specific follicle thyroid models

For the mouse, rat and human models, the calculated *S* values with radioiodine homogeneously distributed within the follicle lumen are shown in Table 1. For the mouse model, the *S* value was 280% and 110% higher for ^131^I than that for ^123^I and ^125^I, respectively. The largest contribution originated from the β particles for ^131^I (88%) and a similar contribution from the AE and CE for ^123^I and ^125^I. For the rat model, the *S* value was 340% and 230% higher for ^131^I than that for ^123^I and ^125^I, respectively. The largest contribution originated from the β particles for ^131^I (90%) and from the CE for ^123^I (63%) and ^125^I (55%). For the human model, the *S* value was 310% and 650% higher for ^131^I than that for ^123^I and ^125^I, respectively. The largest contribution originated from the β particles for ^131^I (93%) and from the CE for ^123^I (87%) and ^125^I (60%).

**Table 1:** S values for radioiodine in the species-specific single follicle models

	**β particles**	**CE**	**AE**	**Total**	**β particles**	**CE**	**AE**
	***S*** **value**	**Relative contribution**	***S*** **value**	**Relative contribution**	***S*** **value**	**Relative contribution**	***S*** **value**
	**(Gy/Bq·s)**	**(%)**	**(Gy/Bq·s)**	**(%)**	**(Gy/Bq·s)**	**(%)**	**(Gy/Bq·s)**
Nucleus ← Lumen							
Mouse							
^123^I	-	-	1.23E-6	47.9	1.33E-6	52.1	2.56E-6
^125^I	-	-	2.49E-6	53.6	2.16E-6	46.4	4.66E-6
^131^I	8.56E-6	88.2	1.07E-6	11.0	7.74E-8	0.8	9.71E-6
Rat							
^123^I	-	-	6.81E-7	62.7	4.06E-7	37.3	1.09E-6
^125^I	-	-	8.05E-7	55.1	6.57E-7	44.9	1.46E-6
^131^I	4.27E-6	89.8	4.63E-7	9.7	2.38E-8	0.5	4.75E-6
Man							
^123^I	-	-	2.20E-7	86.7	3.39E-8	13.3	2.54E-7
^125^I	-	-	8.16E-8	59.8	5.49E-8	40.2	1.37E-7
^131^I	9.60E-7	93.3	6.71E-8	6.5	2.13E-9	0.2	1.03E-6
Nucleus ← Follicle cells							
Mouse							
^123^I	-	-	1.79E-6	10.2	1.58E-5	89.8	1.76E-5
^125^I	-	-	1.24E-5	32.1	2.62E-5	67.9	3.86E-5
^131^I	1.34E-5	86.2	1.25E-6	8.0	9.03E-7	5.8	1.56E-5
Rat							
^123^I	-	-	9.64E-7	13.0	6.47E-6	87.0	7.43E-6
^125^I	-	-	5.18E-6	32.6	1.07E-5	67.4	1.59E-5
^131^I	6.87E-6	87.4	6.23E-7	7.9	3.66E-7	4.7	7.86E-6
Man							
^123^I	-	-	3.05E-7	19.1	1.29E-6	80.9	1.60E-6
^125^I	-	-	1.06E-6	32.9	2.15E-6	67.1	3.21E-6
^131^I	1.76E-6	89.0	1.45E-7	7.3	7.28E-8	3.7	1.98E-6
Nucleus ← Nucleus							
Mouse							
^123^I	-	-	3.52E-4	1.7	2.06E-2	98.3	2.10E-2
^125^I	-	-	1.38E-2	28.5	3.45E-2	71.5	4.83E-2
^131^I	3.48E-3	70.9	2.18E-4	4.4	1.21E-3	24.7	4.91E-3
Rat							
^123^I	-	-	1.58E-4	2.4	6.40E-3	97.6	6.56E-3
^125^I	-	-	4.35E-3	28.9	1.07E-2	71.1	1.51E-2
^131^I	1.52E-3	76.3	9.74E-5	4.9	3.74E-4	18.8	1.99E-3
Man							
^123^I	-	-	9.03E-5	3.1	2.82E-3	96.9	2.91E-3
^125^I	-	-	1.93E-3	29.1	4.70E-3	70.9	6.63E-3
^131^I	8.44E-4	79.4	5.56E-5	5.2	1.63E-4	15.4	1.06E-3

The *S* value and the relative contribution from the non-radiative transitions β particles, Auger electrons (AE) and internal conversion electrons (CE) to the follicle cell nucleus are given for the species-specific models of mouse, rat and man. ^123^I, ^125^I and ^131^I were homogeneously distributed within the follicle lumen, follicle cells and follicle cell nucleus.

For the mouse, rat and human models, the calculated *S* values with radioiodine homogeneously distributed within the follicle cells are shown in Table 1. For the mouse model, the *S* value for ^131^I was 10% and 60% lower than that for ^123^I and ^125^I, respectively. The largest contribution originated from the β particles for ^131^I (86%) and from the AE for ^123^I (90%) and ^125^I (68%). For the rat model, the *S* value for ^131^I was 6% higher and 50% lower than that for ^123^I and ^125^I, respectively. The largest contribution originated from the β particles for ^131^I (87%) and from the AE for ^123^I (87%) and ^125^I (67%). For the human model, the *S* value for ^131^I was 20% higher and 40% lower than that for ^123^I and ^125^I, respectively. The largest contribution originated from the β particles for ^131^I (89%) and from the AE for ^123^I (81%) and ^125^I (67%).

For the mouse, rat and human models, the calculated *S* values with radioiodine homogeneously distributed within the follicle cell nucleus are shown in Table 1. For the mouse model, the *S* value for ^131^I was 80% and 90% lower than that for ^123^I and ^125^I, respectively. The largest contribution originated from the β particles for ^131^I (71%) and from the AE for ^123^I (98%) and ^125^I (72%). For the rat model, the *S* value for ^131^I was 70% and 90% lower than that for ^123^I and ^125^I, respectively. The largest contribution originated from the β particles for ^131^I (76%) and from the AE for ^123^I (98%) and ^125^I (71%). In the human model, the *S* value for ^131^I was 60% and 80% lower than that for ^123^I and ^125^I, respectively. The largest contribution originated from the β particles for ^131^I (79%) and from the AE for ^123^I (97%) and ^125^I (71%).

### Comparison with previously published cellular *S* values

Comparison between the calculated *S* values for ^123^I, ^125^I and ^131^I homogeneously distributed within the follicle cell nuclei with 4, 6 and 8 μm diameter calculated in the present study and previously published cellular *S* values by Goddu and Budinger [[31]] gave the following results: for ^123^I, the previously published *S* values were 1.3%, 2.9% and 2.4% higher, for ^125^I, 0.07%, 0.99% and 0.53% higher and for ^131^I, 2.1%, 1.5% and 0.71% higher, respectively.

### Radioiodine homogeneously distributed within the follicle lumen in human model

**Figure 1:**
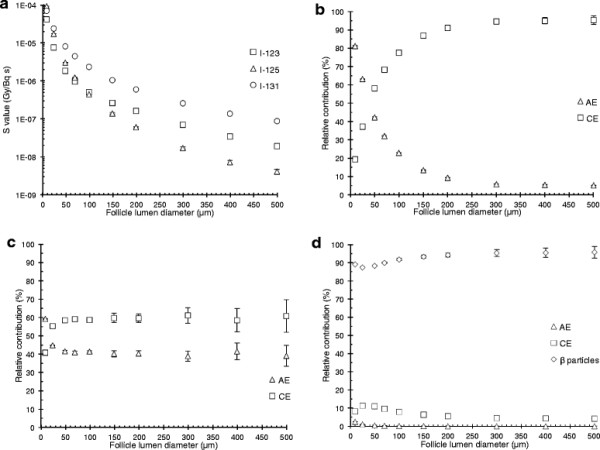
**The** ***S*** **value for homogeneous distribution of radioiodine in the lumen of the human single follicle model.** The *S* value and the relative contributions from the non-radiative transitions for homogeneously distributed radioiodine within the follicle lumen. The follicle lumen diameter varied from 10 to 500 μm surrounded by one single layer of follicle cells with a thickness of 10 μm and centrally placed 8 μm diameter follicle cell nucleus. **(a)** The *S* value for the follicle cell nucleus for ^123^I (square), ^125^I (triangle) and ^131^I (circle), as a function of the follicle lumen diameter. **(b to d)** The relative contribution per decay to the follicle cell nucleus from Auger electrons (AE) (triangle), internal conversion electrons (CE) (square), β particles (diamond), as a function of the follicle lumen diameter, for **(b)**^123^I, **(c)**^125^I and **(d)**^131^I. Error bars indicate the standard deviation of the mean value (SD) and are smaller than the symbol when not visible. Note the logarithmic scale on the ordinate in **(a)**.

For the 8-μm diameter follicle cell nuclei, the calculated *S* values with ^123^I, ^125^I and ^131^I homogeneously distributed within the follicle lumen are shown as a function of the follicle lumen diameter in Figure 1. For the smallest follicle lumen diameter (10 μm), ^125^I gave the highest *S* value, 30% and 120% higher than that for ^131^I and ^123^I, respectively. For larger follicle lumens, ^131^I gave the highest *S* value, 350% and 2,000% higher (500 μm) than that for ^123^I and ^125^I, respectively (Figure 1a). For ^123^I, the relative contributions from AE and CE were 42% and 58% and 5% and 95% for 50-μm and 500-μm lumen diameter, respectively (Figure 1b). For ^125^I, the relative contributions from AE and CE were 42% and 58, and 39% and 61% for 50-μm and 500-μm lumen diameter, respectively (Figure 1c). For ^131^I, the relative contributions from the β particles, AE and CE were 88%, 11% and 0.6% and 96%, 4% and 0.1% for 50-μm and 500-μm lumen diameter, respectively (Figure 1d).

### Radioiodine heterogeneously distributed within the human thyroid follicle model

**Figure 2:**
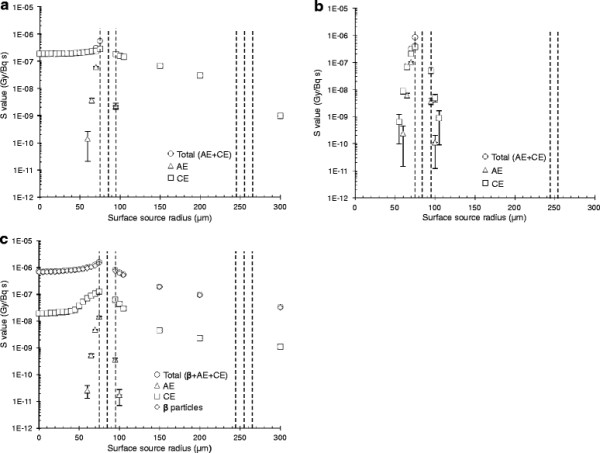
**The** ***S*** **value for heterogeneous distribution of radioiodine in the human follicle model.** The *S* value for the follicle cell nuclei from the non-radiative transitions are given for homogeneously distributed **(a)**^123^I, **(b)**^125^I and **(c)**^131^I on concentric spherical shells in the human thyroid model. The contribution from Auger electrons (AE) (triangle), internal conversion electrons (CE) (square) and β particles (diamond) are shown separately together with the total value (circle). The surface source radius 0 μm indicates the centre of the follicle lumen, and the dashed lines represent the apical and basal follicle cell surfaces for the respective models. Error bars indicate the standard deviation of the mean value (SD) and are smaller than the symbol when not visible. Note the logarithmic scale on the ordinate.

The *S* values were calculated for ^123^I, ^125^I and ^131^I homogeneously distributed on concentric spherical shells in the human model to investigate effects of a heterogeneous distribution (Figure 2). The *S* values were almost constant when ^123^I was situated inside the central follicle lumen due to the CE (Figure 2a). When ^123^I was situated on the apical follicle cell surface, the contributions from the AE and CE were similar and 200% higher than when ^123^I was situated in the centre of the central follicle lumen. The contribution from ^123^I outside the central follicle decreased with increasing radius. When ^125^I was situated in the centre of the follicle lumen, the *S* values were zero until about 20 μm from the apical follicle cell surface due to the short range of emitted AE and CE (Figure 2b). When ^125^I was situated on the apical follicle cell surface, the contribution from the AE was slightly higher, with 56% compared with 44% from the CE. The *S* value increased somewhat when ^131^I was situated peripherally in the lumen compared to that in the centre of the follicle lumen (Figure 2c). The highest contribution originated from the β particles irrespectively of location and was 120% higher when ^131^I was situated on the apical cell surface compared when centrally situated in the follicle lumen. The contribution from ^131^I outside the follicle decreased with increased radius.

### Multiple thyroid follicle models

#### Contribution from surrounding follicle layers in the human model

The calculated *S* values and the relative contributions to the mean absorbed dose to the inner follicle cell nuclei are presented in Table 2 with ^123^I, ^125^I and ^131^I homogeneously distributed within various source compartments (the follicle lumens and cells) in the human multiple thyroid follicle model (*S* values for the mouse and rat models, see Additional file 1). When ^123^I was homogeneously distributed in the entire model, the contribution to the mean absorbed dose from the central follicle was 37%. When ^125^I was homogeneously distributed in the entire model, the contribution to the mean absorbed dose from the central follicle was 90%. Due to the short range of the AE and CE, only one surrounding follicle layer contributed. When ^131^I was homogeneously distributed in the entire model, the contribution to the mean absorbed dose from the central follicle was 11%. The central follicle and eight surrounding follicle layers contributed with approximately 99% of the absorbed dose, with the largest contribution of approximately 30% from the first surrounding follicle layer.

**Table 2:** S values for radioiodine in the human multiple thyroid models

**Source compartment**	***r***_**1**_	***r***_**2**_	***S*** **value**	**Relative contribution**^**a**^	**Relative contribution**^**b**^	**Relative contribution**^**c**^
	**(μm)**	**(μm)**	**(Gy/Bq·s)**	**(%)**	**(%)**	**(%)**
123I						
Lumen 1	0	75	2.54E-07	9.6	15.0	-
Cell layer 1	75	85	1.60E-06	27.6	-	77.3
Cell layer 2a	85	95	2.77E-07	6.1	-	16.9
Lumen layer 2	95	245	4.23E-08	52.7	82.0	-
Cell layer 2b + 3a	245	265	5.86E-09	2.1	-	5.8
Lumen layer 3	265	415	3.97E-10	1.9	2.9	-
Cell layer 3b	415	425	7.50E-12	0.0	-	0.0
				100	100	100
^125^I						
Lumen 1	0	75	1.37E-07	7.7	93.6	-
Cells layer 1	75	85	3.21E-06	82.5	-	89.9
Cells layer 2a	85	95	2.85E-07	9.3	-	10.1
Lumen layer 2	95	245	2.86E-10	0.5	6.4	-
Cell layer 2b	245	255	0.00E + 00	0.0	-	0.0
				100	100	100
^131^I						
Lumen 1	0	75	1.03E-06	5.7	6.9	-
Cells layer 1	75	85	1.98E-06	5.0	-	30.0
Cells layer 2a	85	95	1.02E-06	3.3	-	19.6
Lumen layer 2	95	245	1.44E-07	26.4	31.6	-
Cells layer 2b + 3a	245	265	5.14E-08	2.6	-	15.8
Lumen layer 3	265	415	2.36E-08	16.4	19.7	-
Cells layer 3b + 4a	415	435	1.35E-08	1.9	-	11.6
Lumen layer 4	435	585	7.68E-09	11.9	14.3	-
Cells layer 4b + 5a	585	605	5.39E-09	1.5	-	9.0
Lumen layer 5	605	755	3.28E-09	9.0	10.8	-
Cells layer 5b + 6a	755	775	2.08E-09	1.0	-	5.8
Lumen layer 6	775	925	1.31E-09	5.6	6.8	-
Cells layer 6b + 7a	925	945	9.70E-10	0.7	-	4.0
Lumen layer 7	945	1,095	6.81E-10	4.2	5.1	-
Cells layer 7b + 8a	1,095	1,115	3.67E-10	0.4	-	2.1
Lumen layer 8	1,115	1,265	3.30E-10	2.8	3.3	-
Cells layer 8b + 9a	1,265	1,285	2.21E-10	0.3	-	1.7
Lumen layer 9	1,285	1,435	1.12E-10	1.2	1.5	-
Cell layer 9b	1,435	1,445	6.30E-11	0.1	-	0.3
				100	100	100

The *S* values for the innermost follicle cell nuclei from radioiodine homogeneously distributed in the different source compartments in the human multiple thyroid follicle models (spherical compartments with *r*_1_ = inner radius and *r*_2_ = outer radius). The relative contributions to the mean absorbed dose to the innermost follicle cell nuclei from each compartment are shown for three radionuclide distributions: for ^123^I with two surrounding layers of follicles contributing, ^125^I with one surrounding layer of follicles contributing, and ^131^I with eight surrounding layers of follicles contributing. Follicle layers beyond these contributed with <1%. ^a^Radioiodine homogeneously distributed within the follicle lumens and follicle cells (the entire thyroid model). ^b^Radioiodine homogeneously distributed within the follicle lumens. ^c^Radioiodine homogeneously distributed within the follicle cells.

### Mean absorbed dose for the multiple thyroid follicle model of man – comparison with MIRD formalism

To obtain a mean absorbed dose of 1 Gy to the cell nuclei from homogeneously distributed for ^123^I, ^125^I and ^131^I, in the human multiple follicle model using MIRD formalism and nuclear decay data, the cumulative specific activities $\left(\tilde{C}\left(r_{S},T_{D}\right)\right)$ equal to 234, 378 and 32.6 TBq⋅s/kg, respectively, are needed. Using the same $\tilde{C}\left(r_{S},T_{D}\right)$ in the MC simulations, the mean absorbed dose to the central follicle cell nuclei with radioiodine homogeneously distributed within the follicle lumens and follicle cells was 1.09, 1.18 and 1.04 Gy for ^123^I, ^125^I and ^131^I, respectively, in our multiple thyroid follicle models.

**Figure 3:**
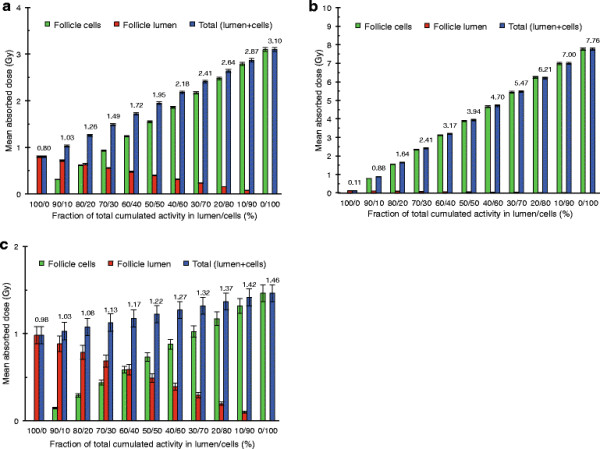
**Mean absorbed dose for different distributions of radioiodine in the human multiple follicle model.** The mean absorbed dose to the central follicle cell nuclei in the human multiple thyroid follicle model for different relations in radioiodine concentration (homogeneous in each compartment) between follicle lumens and/or follicle cells for **(a)**^123^I, **(b)**^125^I and **(c)**^131^I. The values indicate total mean absorbed dose (blue bars, numbers), and the contribution to the mean absorbed dose from the follicle lumen (red bars) and from the follicle cells (green bars). The error bars indicate the standard deviation of the mean value (SD).

The mean absorbed dose to the central follicle cell nuclei for various fractions of radioiodine distributed in the cells versus lumens are shown in Figure 3. The mean absorbed dose was 0.80 Gy ($\tilde{C}\left(r_{S},T_{D}\right)$ = 267 TBq⋅s/kg) when ^123^I was homogeneously distributed within the follicle lumens only, while it was 3.1 Gy ($\tilde{C}\left(r_{S},T_{D}\right)$ = 1,860 TBq⋅s/kg) when ^123^I was located within the follicle cells only. For ^125^I, the corresponding results were 0.11 Gy ($\tilde{C}\left(r_{S},T_{D}\right)$ = 440 TBq⋅s/kg) and 7.8 Gy ($\tilde{C}\left(r_{S},T_{D}\right)$ = 2,700 TBq⋅s/kg), and for ^131^I 0.98 Gy ($\tilde{C}\left(r_{S},T_{D}\right)\;$ = 37 TBq⋅s/kg) and 1.5 Gy ($\tilde{C}\left(r_{S},T_{D}\right)$ = 276 TBq⋅s/kg).

## Discussion

In general, the *S* values determined in the present study were in good agreement with the few corresponding data available in the literature. Our results for ^123^I, ^125^I and ^131^I homogeneously distributed within the follicle cell nuclei in the species-specific models were in excellent agreement with published cellular S values calculated with an analytical method [[31]] based on the experimental range-energy relationship for electrons by Cole [[32]].

The mean absorbed dose to the follicle cells for ^131^I homogeneously distributed within the follicle lumens was determined for a similar human thyroid follicle model as ours [[24]]. Their model included interstitial tissue (51% of the total tissue volume), and 12 surrounding follicles layers, compared with no interstitial tissue and 8 follicle layers in our human model (since we found that the contribution from surrounding layers beyond the 8th was <1%). The contribution from the central follicle was 7% in our model compared with 17% in their model, and the contribution from the surrounding follicles in our model was slightly higher, e.g. 32% for the first surrounding layer compared with 29%. These differences could mainly be explained by the difference in consideration of interstitial tissue. Despite these model differences we found in both studies that the follicle cells received 0.98 Gy when the mean absorbed dose was 1 Gy to thyroid tissue.

There are some simplifications and assumptions made in the calculations, both for the mathematical models and for physical data. The use of unit density water (1.0 g/cm^3^) in the models instead of the density of the thyroid gland, which according to ICRP publication 23 is 1.05 g/cm^3^ [[33]]. Dosimetric calculations performed with a 3% mass concentration of ^127^I (stable iodine) homogenously distributed within the follicle lumens only showed a small difference for ^211^At [[19]], and after normalisation of the mass for the follicle cell nuclei this difference would be even less. Other assumptions regarding the thyroid models have previously been discussed [[19]]. In general, we estimate that the assumptions in the dosimetrical calculations, such as limitations in nuclear decay data and transport physics used by the Monte Carlo code only contribute to a minor extent to the results. The conventional AE spectrum from ICRP 107 was used in the calculations [[26]] and have been regarded adequate when calculating absorbed doses to regions with diameters larger than 1 μm [[34]], and AE with initial kinetic energy lower than 1 keV (the lowest cutoff energy for electrons in MCNPX 2.6.0) were assumed to be fully absorbed within the source volume. This assumption is realistic since experiments have shown that electrons with a kinetic energy of 1 keV have a range of about 61 nm in unit density matter [[32]], verified by calculations: absorbed fraction very close to unity for unit density water spheres with radius of 2 μm for monoenergetic 1 keV electrons [[35]]. Bremsstrahlung generated by the electrons was not accounted for in the Monte Carlo calculations, but the contribution was low in this application (for 1 MeV electrons, only about 0.7% of the kinetic energy is transferred to bremsstrahlung in liquid water, and this fraction is even less for lower kinetic energies [[36]]). The contribution from ^131m^Xe was not considered in the dosimetric calculations for ^131^I, which would result in an underestimation of the *S* value to the cell nucleus of about 3.6% for ^131^I homogenously distributed in an 8-μm cell nucleus and about 1.5% for a homogenous distribution in a 150-μm diameter lumen (unpublished data). Furthermore, the contribution from the xenon daughter is probably less significant due to the short retention in the thyroid gland because of its gaseous state [[23]].

For ^123^I, the contributions from the ^123^Te and ^123m^Te daughters were not included in the dosimetric calculations due to a very long half-life and low yield, respectively. Furthermore, the effects of the charge of the tellurium atoms (average charge of about +9 [[37]] due to multiple ionisation when ^123^I and ^125^I emit cascades of AE) were not considered. Otherwise, such charged atoms may produce ionizations and excitations in the immediate vicinity of the decay site [[37]], which could enhance the biological effect when covalently bound to the DNA [[38]].

With ^123^I and ^125^I homogeneously distributed within the follicle cell nucleus, the *S* value was 2.3 times higher for ^125^I than that for ^123^I in the mouse, rat and human models, a result in accordance with similar calculations for a 10-μm diameter tissue sphere, excluding charge neutralisation [[37]].

Biodistribution studies performed on mice, rats and guinea pigs have shown that the highest uptake of radioiodide was in the thyroid gland, with the highest concentration occurring around 18 to 24 h after injection [[39]-[41]], while the maximal concentration is obtained after approximately 1 to 2 days in normal humans [[42]]. Preclinical studies have shown that radioiodide is rapidly transported through the follicle cell cytoplasm. At early time-points, radioiodine appears as rings peripherally in the follicle lumen close to the apical cell surface [[43]-[46]], and thereafter the radioiodine is more homogeneously distributed in the follicle lumen [[45],[46]]. However peripheral rings have been observed as long as 99 days after injection [[46]]. The specific activity was initially highest in the smallest follicles but became independent of follicle size with time [[45]]. In the human model, the *S* value for ^123^I, ^125^I and ^131^I distributed on the apical follicle cell surface was 2.2, 5.9 and 1.5 times higher than for a homogeneous distribution within the follicle lumen, respectively. Due to the much shorter half-life of ^123^I (13 h), the fraction of decays in the follicle cells and at the apical surface would be highest for ^123^I, indicating a possible higher absorbed dose when biokinetic data are considered.

The MIRD formalism assumes a homogeneous distribution of the radionuclide within the source compartment when determining the mean absorbed dose. Compared with our results, the mean absorbed dose calculated according to MIRD formalism (Equation 1) and nuclear decay data was lower, with the largest difference of 18% for ^125^I, and the smallest of 4% for ^131^I. This comparison together with the dosimetric data obtained for inhomogeneous distribution shows the importance of taking the range of the emitted particles into account. For ^125^I, the emitted low-energy AE and CE, with a range of up to 23 μm in water [[36]], could contribute to a heterogeneous absorbed dose distribution, and about 90% of the absorbed dose originates from the follicle itself. The high-energy β particles emitted by ^131^I, with a range of up to 2.1 mm in water [[24]], contribute to a cross-fire effect with contributions from eight surrounding layers of follicles, which results in a more homogeneous absorbed dose distribution, and only about 11% of the mean absorbed dose originates from the follicle itself. For ^123^I, the emitted low-energetic AE and somewhat higher-energetic CE, the mean absorbed dose was about 9% higher than that according to MIRD formalism. The absorbed dose is then a combination between heterogeneous absorbed dose distribution from the very short-ranged AE and the more homogeneous absorbed dose distribution from the more long-ranged CE, and about 38% of the mean absorbed dose originates from the follicle itself. Furthermore, in the peripheral regions of the thyroid gland where fewer surrounding follicles contribute, the absorbed dose may be lower and more heterogeneous. This effect would be largest for ^131^I with eight surrounding follicle layers contributing and could lead to a reduced mean absorbed dose by up to about 45%.

For radioiodine homogeneously distributed only within the follicle lumens, the mean absorbed dose was 0.80, 0.11 and 0.98 Gy, respectively, for ^123^I, ^125^I and ^131^I, compared with 1 Gy calculated with MIRD formalism and nuclear decay data for radioiodine homogenously distributed within both follicle cells and lumens. Thus, the MIRD formalism overestimates the mean absorbed dose for ^123^I and ^125^I.

## Conclusions

This study clearly demonstrates the importance of using more detailed dosimetric methods and models than MIRD formalism for radioiodine within the thyroid. For radioiodine homogeneously distributed in the follicle cells and lumens, calculations for our human multiple follicle model gave up to 18% higher mean absorbed dose. For radioiodine homogeneously distributed in the follicle lumens only, our calculations gave up to 90% lower mean absorbed dose for ^125^I (20% lower for ^123^I, and 2% lower for ^131^I).

## Competing interests

The authors declare that they have no competing interests.

## Authors’ contributions

AJ designed the study, performed the Monte Carlo simulations and drafted the manuscript. Both authors contributed to the scientific and intellectual discussion and interpretation of the data and revision of the manuscript. Both authors read and approved the final manuscript.

## Authors’ information

AJ is a PhD student in medical science at the Department of Radiation Physics, the Sahlgrenska Academy at the University of Gothenburg. AJ is also a licenced medical physicist. EFA is professor and Head of the Department of Radiation Physics, the Sahlgrenska Academy at the University of Gothenburg and senior medical physicist at Sahlgrenska University Hospital in Gothenburg.

## Additional file

## Supplementary Material

Additional file 1 ***S* values for radioiodine in the mouse and rat multiple thyroid models. Table S1.***S* values for radioiodine in the mouse multiple thyroid models. The *S* values for the innermost follicle cell nuclei from radioiodine homogeneously distributed in the different source compartments in the mouse multiple thyroid follicle models (spherical compartments with *r*_1_ = inner radius and *r*_2_ = outer radius), for ^123^I with five surrounding layers of follicles contributing, ^125^I with one surrounding layer of follicles contributing and ^131^I with ten surrounding layers of follicles contributing. **Table S2.***S* values for radioiodine in the rat multiple thyroid models. The *S* values for the innermost follicle cell nuclei from radioiodine homogeneously distributed in the different source compartments in the rat multiple thyroid follicle models (spherical compartments with r_1_ = inner radius and r_2_ = outer radius), for ^123^I with four layers of follicles contributing, ^125^I with one surrounding layer of follicles contributing and ^131^I with sixteen surrounding layers of follicles contributing. Click here for file
