# Supplementary material for: Dosimetric analysis of 123I, 125I and 131I in thyroid follicle models
Source: EJNMMI Res. 2014 Jun 11;4:23. doi: 10.1186/s13550-014-0023-9 (PMC4078321; doi:10.1186/s13550-014-0023-9)
Supplement: Additional file 1 — S values for radioiodine in the mouse and rat multiple thyroid models. Table S1.S values for radioiodine in the mouse multiple thyroid models. The S values for the innermost follicle cell nuclei from radioiodine homogeneously distributed in the different source compartments in the mouse multiple thyroid follicle models (spherical compartments with r1 = inner radius and r2 = outer radius), for 123I with five surrounding layers of follicles contributing, 125I with one surrounding layer of follicles contributing and 131I with ten surrounding layers of follicles contributing. Table S2.S values for radioiodine in the rat multiple thyroid models. The S values for the innermost follicle cell nuclei from radioiodine homogeneously distributed in the different source compartments in the rat multiple thyroid follicle models (spherical compartments with r1 = inner radius and r2 = outer radius), for 123I with four layers of follicles contributing, 125I with one surrounding layer of follicles contributing and 131I with sixteen surrounding layers of follicles contributing. [file s13550-014-0023-9-S1.docx]

**Table S1. S values for radioiodine in the mouse multiple thyroid models.** The S values for the innermost follicle cell nuclei from radioiodine homogeneously distributed in the different source compartments in the mouse multiple thyroid follicle models (spherical compartments with r_1_=inner radius and r_2_=outer radius), for ^123^I with five surrounding layers of follicles contributing, ^125^I with one surrounding layer of follicles contributing, and ^131^I with ten surrounding layers of follicles contributing.

| Mouse multiple thyroid follicle model | | | | | |
| --- | --- | --- | --- | --- | --- |
| Source compartment | r_1_ (μm) | r_2_ (μm) | ^123^I S value (Gy/Bq⋅s) | ^125^I S value (Gy/Bq⋅s) | ^131^I S value (Gy/Bq⋅s) |
| Lumen 1 | 0 | 25 | 2.56E-06 | 4.66E-06 | 9.71E-06 |
| Cell layer 1 | 25 | 31 | 1.76E-05 | 3.86E-05 | 1.56E-05 |
| Cell layer 2a | 31 | 37 | 2.38E-06 | 4.29E-06 | 7.21E-06 |
| Lumen layer 2 | 37 | 87 | 2.76E-07 | 5.08E-08 | 1.32E-06 |
| Cell layer 2b (only ^125^I) | 87 | 93 | - | 0.00E+00 | - |
| Cells layer 2b+3a | 87 | 99 | 1.59E-07 | - | 5.05E-07 |
| Lumen layer 3 | 99 | 149 | 1.10E-07 | - | 2.57E-07 |
| Cells layer 3b+4a | 149 | 161 | 7.58E-08 | - | 1.53E-07 |
| Lumen layer 4 | 161 | 211 | 3.41E-08 | - | 1.02E-07 |
| Cells layer 4b+5a | 211 | 223 | 1.07E-08 | - | 7.41E-08 |
| Lumen layer 5 | 223 | 273 | 3.00E-09 | - | 5.13E-08 |
| Cells layer 5b+6a | 273 | 285 | 9.97E-10 | - | 4.05E-08 |
| Lumen layer 6 | 285 | 335 | 1.38E-10 | - | 3.03E-08 |
| Cell layer 6b (only ^123^I) | 335 | 341 | 4.62E-11 | - | - |
| Cells layer 6b+7a | 335 | 347 | - | - | 2.60E-08 |
| Lumen layer 7 | 347 | 397 | - | - | 1.97E-08 |
| Cells layer 7b+8a | 397 | 409 | - | - | 1.53E-08 |
| Lumen layer 8 | 409 | 459 | - | - | 1.33E-08 |
| Cells layer 8b+9a | 459 | 471 | - | - | 1.16E-08 |
| Lumen layer 9 | 471 | 521 | - | - | 8.46E-09 |
| Cells layer 9b+10a | 521 | 533 | - | - | 6.68E-09 |
| Lumen layer 10 | 533 | 583 | - | - | 6.21E-09 |
| Cells layer 10b+11a | 583 | 595 | - | - | 4.62E-09 |
| Lumen layer 11 | 595 | 645 | - | - | 3.78E-09 |
| Cell layer 11b (only ^131^I) | 645 | 651 | - | - | 4.37E-09 |

**Table S2. S values for radioiodine in the rat multiple thyroid models.** The S values for the innermost follicle cell nuclei from radioiodine homogeneously distributed in the different source compartments in the rat multiple thyroid follicle models (spherical compartments with r_1_=inner radius and r_2_=outer radius), for ^123^I with four layers of follicles contributing, ^125^I with one surrounding layer of follicles contributing, and ^131^I with sixteen surrounding layers of follicles contributing.

| Rat multiple thyroid follicle model | | | | | |
| --- | --- | --- | --- | --- | --- |
| Source compartment | r_1_ (μm) | r_2_ (μm) | ^123^I S value (Gy/Bq⋅s) | ^125^I S value (Gy/Bq⋅s) | ^131^I S value (Gy/Bq⋅s) |
| Lumen 1 | 0 | 35 | 1.09E-06 | 1.46E-06 | 4.75E-06 |
| Cell layer 1 | 35 | 43 | 7.43E-06 | 1.59E-05 | 7.86E-06 |
| Cell layer 2a | 43 | 51 | 9.84E-07 | 1.57E-06 | 3.67E-06 |
| Lumen layer 2 | 51 | 121 | 1.72E-07 | 7.09E-09 | 6.57E-06 |
| Cell layer 2b (only ^125^I) | 121 | 129 | - | 0.00E+00 | - |
| Cells layer 2b+3a | 121 | 137 | 1.04E-07 | - | 2.53E-07 |
| Lumen layer 3 | 137 | 207 | 4.82E-08 | - | 1.25E-07 |
| Cells layer 3b+4a | 207 | 223 | 1.29E-08 | - | 7.36E-08 |
| Lumen layer 4 | 223 | 293 | 2.73E-09 | - | 4.80E-08 |
| Cells layer 4b+5a | 293 | 309 | 3.89E-10 | - | 3.40E-08 |
| Lumen layer 5 | 309 | 379 | 9.21E-11 | - | 2.29E-08 |
| Cell layer 5b (only ^123^I) | 379 | 387 | 1.44E-11 | - | - |
| Cells layer 5b+6a | 379 | 395 | - | - | 1.64E-08 |
| Lumen layer 6 | 395 | 465 | - | - | 1.29E-08 |
| Cells layer 6b+7a | 465 | 481 | - | - | 9.42E-09 |
| Lumen layer 7 | 481 | 551 | - | - | 7.70E-09 |
| Cells layer 7b+8a | 551 | 567 | - | - | 6.49E-09 |
| Lumen layer 8 | 567 | 637 | - | - | 5.24E-09 |
| Cells layer 8b+9a | 637 | 653 | - | - | 4.07E-09 |
| Lumen layer 9 | 653 | 723 | - | - | 3.43E-09 |
| Cells layer 9b+10a | 723 | 739 | - | - | 2.81E-09 |
| Lumen layer 10 | 739 | 809 | - | - | 2.09E-09 |
| Cells layer 10b+11a | 809 | 825 | - | - | 1.50E-09 |
| Lumen layer 11 | 825 | 895 | - | - | 1.50E-09 |
| Cells layer 11b+12a | 895 | 911 | - | - | 1.38E-09 |
| Lumen layer 12 | 911 | 981 | - | - | 1.03E-09 |
| Cells layer 12b+13a | 981 | 997 | - | - | 7.28E-10 |
| Lumen layer 13 | 997 | 1067 | - | - | 5.73E-10 |
| Cells layer 13b+14a | 1067 | 1083 | - | - | 4.30E-10 |
| Lumen layer 14 | 1083 | 1153 | - | - | 3.19E-10 |
| Cells layer 14b+15a | 1153 | 1169 | - | - | 3.52E-10 |
| Lumen layer 15 | 1169 | 1239 | - | - | 2.42E-10 |
| Cells layer 15b+16a | 1239 | 1255 | - | - | 1.76E-10 |
| Lumen layer 16 | 1255 | 1325 | - | - | 1.71E-10 |
| Cells layer 16b+17a | 1325 | 1341 | - | - | 2.29E-10 |
| Lumen layer 17 | 1341 | 1411 | - | - | 6.60E-10 |
| Cell layer 17b (only ^131^I) | 1411 | 1419 | - | - | 7.73E-11 |
